# Effect of teriparatide (rh-PTH 1–34) versus bisphosphonate on the healing of osteoporotic vertebral compression fracture: A retrospective comparative study

**DOI:** 10.1186/s12891-017-1509-1

**Published:** 2017-04-07

**Authors:** Akira Iwata, Masahiro Kanayama, Fumihiro Oha, Tomoyuki Hashimoto, Norimasa Iwasaki

**Affiliations:** 1 Spine Center, Hakodate Central General Hospital, Hon-cho 33-2, Hakodate, Hokkaido 040-8585 Japan; 2 Department of Orthopaedic Surgery, Hokkaido University, N15 W7 Kita-ward, Sapporo, Hokkaido 060-8638 Japan

**Keywords:** Teriparatide, Recombinant human parathyroid hormone 1–34, Bisphosphonates, Union rate, Osteoporosis, Osteoporotic vertebral compression fracture

## Abstract

**Background:**

Teriparatide (recombinant human parathyroid hormone 1–34) is increasingly used for the treatment of severe osteoporosis because it stimulates bone formation and may potentially enhance fracture healing. The objective of this study was to investigate the effects of teriparatide versus a bisphosphonate on radiographic outcomes in the treatment of osteoporotic vertebral compression fractures (OVCF).

**Methods:**

A total of 98 patients undergoing non-operative treatment for recent single-level OVCF were reviewed retrospectively. Thirty-eight patients were treated by a once-daily subcutaneous injection of 20 micrograms of teriparatide (TPD group), whereas 60 patients received 35 mg of alendronate weekly (BP group). Except for these medications, the same treatment protocol was applied to both groups. The radiographic assessments included union status, vertebral kyphosis, and mid-vertebral body height. The rates of fracture site surgical intervention were also compared between the two groups. The mean follow-up period was 27 months (median 22.5, range 2 – 75 months).

**Results:**

Cox regression analysis showed that TPD reduced the time-to-union (adjusted relative hazard ratio: 1.86, 95% C.I.: 1.21 – 2.83). The union rate at six months after treatment was 89% in the TPD group and 68% in the BP group; the surgical intervention rate was significantly higher in the TPD group (*p* = 0.026, adjusted odds ratio: 8.15, 95% C.I.: 2.02 – 43.33). The change in local kyphosis was 4.6° in the TPD group and 3.8° in the BP group (*p* = 0.495, paired *t*-test). The change of mid-vertebral body height was 4.4 mm in the TPD group and 3.4 mm in the BP group (*p* = 0.228, paired *t*-test). Fracture site surgical interventions were not required in the TPD group; however, two patients in the BP group eventually underwent surgical treatment for symptomatic non-union or vertebral collapse.

**Conclusions:**

This retrospective study suggests that teriparatide may enhance fracture healing and improve the union rate in OVCF.

## Background

Pharmaceutical agents play a major role in the treatment of osteoporosis. Bisphosphonates, which inhibit osteoclast activity and bone resorption, are the current first-line medications for osteoporosis [1]. The robust suppression of osteoclast activity may hinder bone remodeling and maturation in the fracture healing process [2]. Although this adverse effect on fracture healing might raise concerns, such potential effects on healing have not been clinically evidenced in a meta-analysis of prospective randomized trials [3] and a systematic review [4].

Teriparatide (TPD, recombinant human parathyroid hormone (PTH) 1–34), an osteogenic osteoporosis agent, has been used increasingly for patients with severe osteoporosis and at high risk of fractures. Intermittent systemic administration of PTH induces bone formation through stimulation of osteoblast proliferation [5], prevention of osteoblast apoptosis [6] and increased osteoblast activity [5, 7]. This pharmaceutical agent may potentially enhance fracture healing.

Failure of fracture healing in osteoporotic vertebral compression fracture (OVCF) leads to intractable back pain associated with non-union [8, 9] and delayed vertebral collapse, resulting in neurological deficits [10]. Percutaneous vertebral augmentation is a widely accepted treatment option for OVCF. In particular, balloon kyphoplasty (BKP) may correct vertebral body height and local kyphotic deformity [11]. However, the differences in pain control between patients who received non-surgical care and kyphoplasty treatment were diminished at 12 months because the non-surgical group improved over time, probably as a result of fracture healing [12, 13]. Differences in the eventual outcome of patients with OVCF depend greatly on the union status. To date, however, whether teriparatide may enhance OVCF healing remains unclear. The current retrospective comparative study aimed to investigate the effect of teriparatide versus bisphosphonate on radiographic outcomes in the non-operative treatment of OVCF and to determine whether teriparatide enhances OVCF fracture healing.

### Methods

A total of 98 patients who underwent non-operative treatment for a single-level recent OVCF between April 2010 and March 2012 were reviewed retrospectively. Included were 12 males and 86 females with a mean age of 76.7 years (58 – 94 years). A recent OVCF was diagnosed using 1.5-tesla MRI when a signal change of the affected vertebra (low signal intensity on the T1-weighted image or high signal intensity in short inversion time inversion recovery on the T2-weighted image) was observed. The bone mineral densities of the lumbar spine and femoral neck were measured before treatment using dual-energy X-ray absorptiometry (DXA; Delphi QDR System, Toyo Medic Company, Tokyo, Japan). Osteoporosis was defined by a DXA-measured lumbar spine or femoral neck bone mineral density below 70% of the young adult mean (YAM) (T-score less than −2.5) or 70-80% of YAM (T-score under −2.5 to −1.5) with a fragility-related fracture [14]. Patients with steroid-induced osteoporosis or those who underwent dialysis for chronic renal failure were excluded from the current investigation. The patients were divided into two treatment groups based on the medication used: 38 patients received teriparatide (TPD group) and 60 patients received a bisphosphonate (BP group). Background demographic data are listed in Table 1.

**Table 1:** Demographic data for the teriparatide group versus the bisphosphonate group

	TPD (n = 38)	BP (*n* = 60)	*p*-value
Age (years) (mean ± SD)	75.5 ± 7.1	77.6 ± 8.0	0.205
Gender (male:female)	4:34	8:52	0.761
BMD at lumbar spine (g/cm^2^) (mean ± SD)	0.709 ± 0.020	0.692 ± 0.019	0.561
BMD at femoral neck (g/cm^2^) (mean ± SD)	0.520 ± 0.018	0.531 ± 0.015	0.655
GFR (mL/min/1.73^2^)	64.0 ± 3.2	65.1 ± 2.8	0.796
Pre-existing VCF (present: not present)	8:30	15:45	0.653
Level of vertebral fracture (non-TL:TL)	17:21	18:42	0.138
Posterior wall fracture (not injured:injured)	16:22	28:32	0.658
Prior bisphosphonate use (not used:used)	17:21	42:18	0.021*

*p < 0.05, *TPD* teriparatide group, *BP* bisphosphonate group, *SD* standard deviation, *BMD* bone mineral density, *GFR* glomerular filtration rate, *VCF* vertebral compression fracture, *TL* thoracolumbar spine (T11-L2), *non-TL* non-thoracolumbar spine (T5-T10 or L3-L5)

The TPD group received 20 micrograms of teriparatide administered by subcutaneous injection once daily. In the BP group, 35 mg of alendronate was orally administered once a week (the therapeutic dose of alendronate, 70 mg per week in the United States and Europe, is limited to 35 mg per week in Japan). The bisphosphonate group consisted of patients with OVCF who had been treated with alendronate in the same manner from 2006. The TPD group consisted of those who were treated using teriparatide after teriparatide became available in Japan. No patients in either group received calcium or vitamin D supplements. Except for TPD and BP administration, the same treatment protocol was applied to both groups. Patients were permitted to ambulate with a custom-made plastic orthosis during the first week of treatment. The mean follow-up period was 27 months (median 22.5, range 2 to 75 months). Fifteen patients in the TPD group and 10 patients in the BP group completed follow-up within 6 months because radiographic union was achieved.

**Fig. 1:**
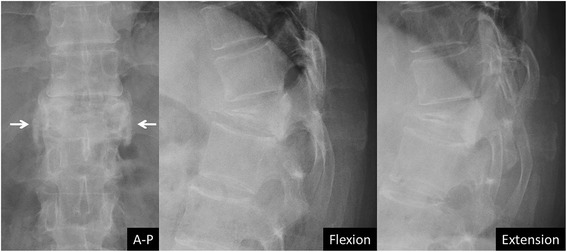
Bridging bone around a vertebral cleft. Although a vertebral cleft was observed, the presence of bony bridging around the fractured vertebra (arrows) was interpreted as diagnostic of radiographic union

Union status and deformity of the fractured vertebra were assessed radiographically. Radiographic examinations were performed once a week during the first month of treatment, once a month until the third month, and once every three months subsequently until union was confirmed. Assessment at six months after treatment was based on the radiographs obtained at six months plus/minus one month. The parameters included union rate at six months and at the final follow-up after treatment, vertebral kyphosis and mid-vertebral body height. Three independent observers assessed radiographic union (two examiners performed the evaluations independently, and another examiner independently assessed the cases when the conclusions of the two observers differed). Non-union was diagnosed by the presence of a vertebral cleft or abnormal motion at the fractured vertebra on the flexion-extension radiographs. Notwithstanding evidence of a vertebral cleft, the presence of trabecular continuity or bridging of bone around the cleft were regarded as radiographic union (Fig. 1). The Kappa statistic of intraclass correlation coefficient was 0.787 (95% confidence interval: 0.674 – 0.901). The Kappa statistic of inter-rater reliability was 0.952 (95% confidence interval: 0.887 – 1.000). The rate of fracture site surgical intervention was also compared between the two groups.

Age and bone mineral density were compared as continuous variables using the unpaired *t*-test. Gender, prevalence of pre-existing vertebral fracture, rate of thoracolumbar (T11-L2) vertebral fracture, incidence of posterior wall fracture, rate of prior bisphosphonate use, and union status (presence or absence of radiographic union) were analyzed as categorical data using Fisher’s exact test. Mid-vertebral body height and kyphosis angle were compared pre- and post-treatment using the paired *t*-test. Progression of vertebral deformity was defined as >15% loss of vertebral body height or >10° of kyphosis progression [15]. The level of significance was defined as a p-value of less than 0.05. Statistical analyses were performed using JMP Pro 11 software (SAS Institute Inc., Cary, North Carolina, USA). Multivariate logistic regression analysis was used to evaluate associations between union status and demographic data including pharmaceutical agents. Multiple dichotomous variables included 1) age (<80 or ≥80 years), 2) gender (male or female), 3) lumbar spine bone mineral density (≤ − 2.5 or > −2.5 SD), 4) fracture level (thoracolumbar (T11-L2) or non-thoracolumbar (T5-T10 or L3-L5)), 5) presence of pre-existing vertebral fracture (existent or non-existent), 6) incidence of posterior wall fracture (injured or not injured), 7) rate of past bisphosphonate use (used or not used), and 8) pharmaceutical agent (TPD or BP). Kaplan-Meier analysis was used to compare the time-to-union between the TPD and BP groups, and Cox regression analysis was used to examine the covariates for time-to-union.

## Results

**Fig. 2:**
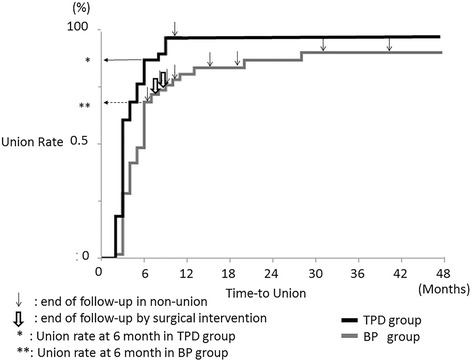
Kaplan-Meier curves for time-to-union in the TPD group versus the BP group. The Kaplan-Meier curve showed the time course of union in the TPD group and the BP group (*p* < 0.001, log-rank test). The thin black arrow shows the end of follow-up with non-union, and the bold white arrow indicates the end of follow-up due to surgical intervention. The union rate was 89% in the TPD group and 68% in the BP group by six months after treatment (*p* = 0.026, Fisher’s exact test). At the final follow-up, 97% of those in the TPD group and 90% of those in the BP group achieved a stable union (*p* = 0.243, Fisher’s exact test)

Kaplan-Meier survival analysis showed a significant difference in the time-to-union between the two groups (*p* < 0.001, log-rank test) (Fig. 2). Cox regression analysis showed an adjusted relative hazard for TPD versus BP use of 1.86 (95% C.I.: 1.21–2.83). The radiographic union rate was 89% (34/38 patients) in the TPD group versus 68% (41/60 patients) in the BP group at six months after treatment, and the difference was significant (*p* = 0.026, Fisher’s exact test). At the final follow-up, 97% of patients (37/38 patients) in the TPD group and 90% of patients (54/60 patients) in the BP group achieved radiographic union (*p* = 0.243, Fisher’s exact test). Multiple logistic regression analyses were conducted using stepwise regression to adjust for heterogeneity (age, gender, fracture level, bone mineral density, pre-existing vertebral fracture, medication used, posterior wall fracture, and prior bisphosphonate use) between the two groups (Table 2). Regarding TPD versus BP use, the adjusted odds ratio for union at six months after treatment was 8.15 (95% C.I.: 2.02–43.33). Fracture site surgical interventions were not required in the TPD group, whereas two patients in the BP group eventually underwent surgical treatment. One patient was treated by balloon kyphoplasty for symptomatic non-union, and the other underwent posterior decompression and fusion for vertebral collapse with a neurological deficit.

**Table 2:** Multivariable logistic regression analysis using stepwise method for union status at 6 months after treatment

Factors	*p*-value	Adjusted odds ratio (95% CI)
Age (<80 vs. ≥80 years)	0.205	2.21 (0.65 – 8.11)
Gender	*p* > 0.25	
Fracture level (TL vs. non-TL)	0.001*	11.67 (2.48 – 83.17)
BMD at lumbar spine (≤ − 2.5 vs. > − 2.5 SD)	0.001*	11.68 (2.48 – 83.17)
Pre-existing VCF	*p* > 0.25	
Posterior wall fracture	0.001*	8.53 (2.25 – 42.23)
Past bisphosphonate use	0.019*	4.86 (1.29 – 22.09)
Pharmaceutical agents (TPD vs. BP)	0.002*	8.15 (2.02 – 43.33)

*p < 0.05, *95% CI* 95% confidence interval, *BMD* bone mineral density, *SD* standard deviation, *VCF* vertebral compression fracture, *TL* thoracolumbar spine (T11-L2), *non-TL* non-thoracolumbar spine (T5-T10 or L3-L5), *TPD* teriparatide, *BP* bisphosphonatecme

**Fig. 3:**
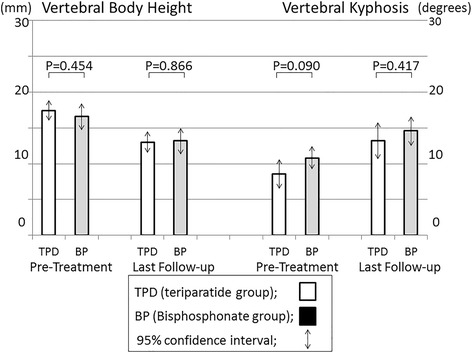
Mid-vertebral body height and vertebral kyphosis angle. Vertebral body height was 17.4 ± 0.7 mm in the TPD group and 16.6 ± 0.9 mm in the BP group before treatment. This measure decreased to 13.0 ± 0.7 mm and 13.2 ± 0.9 mm at the final follow-up, respectively (*p* = 0.228, paired *t*-test). The vertebral kyphosis angle was 8.6 ± 1.0° in the TPD group and 10.8 ± 0.8° in the BP group before treatment. This angle increased to 13.2 ± 1.3° and 14.6 ± 1.0° at the final follow-up, respectively (*p* = 0.495, paired *t*-test)

**Fig. 4:**
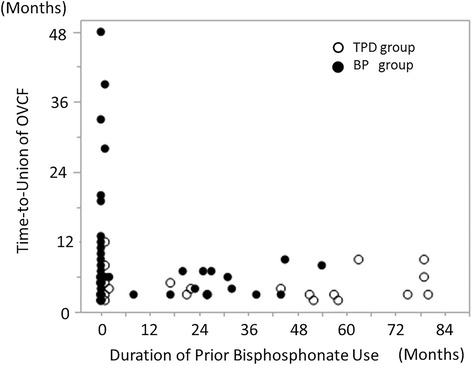
Duration of prior bisphosphonate use and time-to-union of osteoporotic vertebral fracture. This graph showed the relationship between the duration of bisphosphonate use at the time of OVCF and the OVCF time-to-union. The white round mark indicates the TPD group and the black round mark indicates the BP group. The duration of bisphosphonate use did not correlate with OVCF time-to-union in either group

The change of mid-vertebral body height was 4.4 mm in the TPD group and 3.4 mm in the BP group (*p* = 0.228, paired *t*-test) (Fig. 4). The change of local kyphosis was 4.6° in the TPD group and 3.8° in the BP group (*p* = 0.495, paired *t*-test) (Fig. 3). Progression of vertebral deformity (>15% loss of vertebral body height or >10° of kyphosis progression) was observed in 66% of the TPD group (25/38 patients) versus 60% of the BP group (36/60 patients), a difference that was not significant (*p* = 0.670, Fisher’s exact test). The union rate was 84% (21/25) in the TPD group, and 61% (22/36) in the BP group (*p* = 0.086, Fisher’s exact test). Multivariate logistic regression analysis using the stepwise method showed that teriparatide significantly was associated with an increased union rate; the adjusted odds ratio was 7.80 (95% C.I.: 1.41–70.35) in cases with vertebral deformity progression.

Union at six months was achieved in 61% (11/18 patients) of those with prior bisphosphonate use (past-BP) and in 71% (30/42 patients) of those without prior BP use in the BP group (*p* = 0.547, Fisher’s exact test). The union rate was 86% (18/21 patients) among those with prior BP use and 94% (16/17 patients) among those without prior BP use in the TPD group (*p* = 0.613, Fisher’s exact test). The union rate at final follow-up was 89% (16/18 patients) among those with prior BP use and 90% (38/42 patients) among those without prior BP use in the BP group (*p* = 1.000, Fisher’s exact test). Union occurred in 95% (20/21 patients) of those with prior BP use and in 100% (17/17 patients) of those without prior BP use in the TPD group (*p* = 1.000, Fisher’s exact test). Time-to-union significantly differed between the TPD group without prior BP use (3.4 ± 1.8 months) and the BP group without prior BP use (7.7 ± 1.1 months) (*p* = 0.042, *t*-test). Whereas prior bisphosphonate use tended to delay the time-to-union in the TPD group (3.4 ± 0.6 to 4.8 ± 0.5 months) (*p* = 0.070, *t*-test), it did not affect the time-to-union in the BP group (7.8 ± 1.4 to 8.3 ± 2.1 months) (*p* = 0.827) (Table 3). The duration of prior-BP use did not show a correlation with the OVCF time-to-union (Fig. 4).

**Table 3:** Effect of bisphosphonate use prior to osteoporotic vertebral fracture

	With prior BP	Without prior BP	*p*-value
BP group			
Union rate at 6 months	61.0% (11/18)	71.4% (30/42)	0.547
Union rate at final follow-up	88.9% (16/18)	90.5% (38/42)	1.000
Time-to-union (months)	8.3 ± 2.1	7.8 ± 1.4	0.827
	(95% C.I.: 4.2 – 12.5)	(95%C.I.: 5.0 – 10.5)	
TPD group			
Union rate at 6 months	85.7% (18/21)	94.1% (16/17)	0.613
Union rate at final follow-up	95.2% (20/21)	100% (17/17)	1.000
Time-to-union (months)	4.8 ± 0.5	3.4 ± 0.6	0.070
	(95% C.I.: 3.8 – 5.8)	(95% C.I.: 2.3 – 4.5)	
*p*-value			
Union rate at 6 months	0.141	0.084	
Union rate at final follow-up	0.586	0.314	
Time-to-union	0.115	0.042	

*p < 0.05, With prior BP: with bisphosphonate use prior to the time of osteoporotic vertebral fracture, Without prior BP: without bisphosphonate use prior to the time of osteoporotic vertebral fracture

## Discussion

The current study demonstrated that the radiographic union rate by six months after treatment was significantly higher in the teriparatide group (89%) than in the bisphosphonate group (68%), with an adjusted odds ratio of 8.15 (95% C.I.: 2.02–43.33). The time-to-union was significantly less in the teriparatide group (*p* < 0.001, log-rank test). The adjusted relative hazard for TPD versus BP use was 1.78 (95% C.I.: 1.16–2.71). Thus, teriparatide had the advantage of achieving a significantly higher union rate at six months, and of achieving union earlier in the OVCF delayed union stage. Although teriparatide did not contribute to preventing the progression of vertebral deformity, it had the advantage of achieving union, including in cases with vertebral deformity progression (adjusted odds ratio: 7.80, 95% C.I.: 1.41–70.35). This higher union rate despite the progression of vertebral deformity suggests that teriparatide use may help reduce the need for surgical intervention for OVCF.

Previous randomized controlled trials demonstrated that teriparatide accelerated fracture healing. Aspenberg et al. conducted a prospective, randomized, double-blind study of 102 postmenopausal women with distal radius fractures, and showed that the time to cortical bridging was significantly shorter in the once-daily 20-microgram teriparatide group than in the placebo control group [16]. Peichl et al. also demonstrated in their randomized controlled trial of 65 patients with osteoporotic pubic bone fractures that a once-daily injection of PTH 1–84 accelerated healing compared with treatment with vitamin D with calcium [17]. Additionally, several clinical cases were reported to show successful repair of non-union in sternum fractures [18] and dens fractures [19].

According to population-based epidemiologic studies, the prevalence of postmenopausal osteoporosis is approximately 30 to 45% in women aged 70 years [20, 21] and 40–45% in those aged 80 years [21, 22]. Osteoporotic fractures and related problems impact the quality of life in the older adult population. Failure of fracture healing in OVCF leads to intractable back pain associated with non-union [8, 9]. Patients with symptomatic vertebral non-union frequently require surgical interventions, including balloon kyphoplasty or spinal reconstruction. Furthermore, once non-union advances to osteonecrosis, the collapsed vertebrae cause a progressive kyphotic deformity and severe neural tissue compression with neurological deficits [8, 9, 23]. These pathologies can be managed only through major spinal reconstruction using instrumentation [24, 25]. Thus, the utmost priority in the treatment of OVCF is to prevent vertebral non-union and osteonecrosis.

In the current study, the risk factors for non-union were thoracolumbar fracture, decreased bone mineral density, posterior wall fracture, and prior bisphosphonate use. Vertebral non-union typically has been reported to occur mainly in the thoracolumbar zone [9, 13]. This phenomenon occurs due to the high stresses in this region generated by the transition from the rigid thoracic spine to the flexible lumbar spine along with the anterior element force transmission from the kyphotic thoracic spine to the relatively neutral thoracolumbar junction. Bone mineral density was negatively correlated with the intravertebral vacuum occurrence rate [26, 27]. Posterior wall fracture was reported to be a risk factor for progression of vertebral collapse [15, 28]. These factors might be considered related to the vulnerability of the small blood-supplying arteries in the vertebral bodies. Interruption of the blood supply and insufficient bone marrow neovascularization are the likely causes of avascular necrosis of the vertebral body, which may result in vertebral non-union [10, 13, 29]. Once vertebral necrosis occurs, union is difficult to achieve in the presence of a vertebral cleft. Peripheral bridging bone formation around an OVCF with vertebral clefting was often observed using teriparatide, as shown in Fig. 1. With peripheral bridging bone cross-linkage along the vertebral edges, the stability of the collapsed vertebra was recovered. Teriparatide was reported to promote ossification of the spinal ligaments [30] and to accelerate hyperplastic bone formation around vertebral fractures in a case of diffuse idiopathic skeletal hyperostosis [31]. In the current study, teriparatide significantly affected union in cases of vertebral deformity progression (adjusted odds ratio 7.80). Moreover, prior bisphosphonate use was an independent risk factor for non-union at six months. The substantial suppression of osteoclast activity with BP use might be implicated in the current results. Prior bisphosphonate use was associated with delayed union, particularly with teriparatide use. However, the duration of prior bisphosphonate use did not correlate with the time-to-union and the union rate of OVCF at the final follow-up. An explanation for this finding might be that bone metabolism after introducing the bisphosphonate may subside to a constant value by three months, with maintenance of that rate thereafter. Nenonen showed a rapid decrease and persistent change of bone metabolic markers after introducing a bisphosphonate [32]. Once the metabolism was suppressed by the bisphosphonate, bone formation would not ensue with the use of teriparatide.

Both the teriparatide and bisphosphonate groups were similar regarding the inhibition of vertebral deformity progression. Fractured vertebrae require several months to achieve adequate mechanical strength and a stable union. Angular kyphosis develops within several weeks or months after injury [33, 34]. Although teriparatide has the potential to increase bone mineral density and vertebral body strength [35, 36], vertebral deformity might progress before the medication has elicited its effects on vertebral mechanical strength.

Several limitations of the current study should be addressed. This is a retrospective comparative study that included different sample sizes and demographics between the two treatment groups. The inter-group difference in the ratio of prior bisphosphonate use was a potential source of selection bias in this study. To adjust for heterogeneity between the two treatment groups, we used multiple logistic regression analysis. The biological aspects of fracture healing should be examined prospectively using bone metabolic markers, vitamin D, and parathyroid hormone level status to determine whether the state of bone turnover was comparable between the TPD group and the BP group at baseline and to probe whether treatment affected bone turnover in these patients. Due to the nature of retrospective investigation, serum bone markers could be collected only in a very limited number of patients. In this retrospective study, the follow-up duration after three months following treatment differed for each doctor. Considering the follow-up duration differences, we showed the union rate on the Kaplan-Meier survival curve as the time course of vertebral union for each medication. An MRI study might help assess the early phase of fracture healing.

## Conclusions

This retrospective study suggests that teriparatide may potentially enhance fracture healing and improve the union rate in OVCF.

## Abbreviations

95% C.I.: 95% confidence interval
BMD: Bone mineral density
DXA: Dual-energy X-ray absorptiometry
GFR: Glomerular filtration rate
non-TL: Non-thoracolumbar spine (T5-T10 or L3-L5)
OVCF: Osteoporotic vertebral compression fracture
Prior-BP: Bisphosphonate use prior to osteoporotic vertebral compression fracture
PTH: Parathyroid hormone
SD: Standard deviation
TL: Thoracolumbar spine (T11-L2)
TPD: Teriparatide
YAM: Young adult mean value
